# Pre-injury health status of truck drivers with a workers’ compensation claim

**DOI:** 10.1186/s12889-022-13885-4

**Published:** 2022-09-05

**Authors:** Angela Batson, Janneke Berecki-Gisolf, Sharon Newnam, Voula Stathakis

**Affiliations:** 1 Monash University Accident Research Centre, Monash University, 21 Alliance Lane, VIC, 3800 Australia; 2 Queensland University of Technology, School of Psychology and Counselling, VIC, Australia

**Keywords:** Truck driver health, Work injury, Health service use, Occupational health, Occupational drivers, Road environment

## Abstract

Truck drivers are a vulnerable population due to the high number of workplace injuries and fatalities predominant in their occupation. In Australia, the road freight transportation industry has been identified as a national priority area in terms of creating preventative measures to improve the health and safety of its workers. With an environment conducive to poor nutritional food choices and unhealthy lifestyle behaviours, many barriers exist to creating a safe and healthy workforce. Thus, the current study aimed to describe the pre-injury hospital-recorded health conditions and health service use of truck drivers with a worker’s injury compensation claim/s when compared to workers in other industries. Data was obtained from a compensation claims database and linked with hospital admissions data recorded five years prior to the injury claim. Health and lifestyle behaviour data for the occupational code of truck drivers was compared to other occupational drivers, as well as to all other occupations. Analysis was conducted via logistic regression. The results found that when compared to other occupational drivers, truck drivers were significantly more likely to have a hospital-recorded diagnosis of diabetes and/or hypertension, as well as being significantly more likely to have a hospital record of tobacco use and/or alcohol misuse/abuse. The findings show that there is a need to review and revise existing health strategies to promote the health and wellbeing of truck drivers, especially given their challenging work environment.

## Background

Safe Work Australia has identified truck driving as a priority area for health and safety reform due to the high number of fatalities, injuries and illnesses that occurs in this occupational group [1]. Truck drivers are at greater risk of work-related injury and disease even compared with other groups of drivers (i.e., bus drivers, automobile drivers, delivery drivers, rail drivers), with an elevated rate of 70.3 claims per 1000 workers per year [2]. Additionally, the relative risk of workers’ compensation claims increases with age [3]. These statistics suggest that truck driving requires the development and implementation of injury control measures across all levels of the road freight transportation system [4–6]. To achieve this goal, it is critical to identify feasible and practicable solutions. In doing this, it is firstly important to consider the context of the work role.

The work environment has been described as a “healthy food desert” [7.]. Even truck drivers who participate in a healthy lifestyle outside work, can find it difficult to maintain healthy eating behaviours whilst on the road [8]. Prolonged work hours in the driving seat mean that drivers have limited time opportunities for being active and for seeking healthy meal options [9]. A focus group of long-haul truck drivers reported that despite a desire to eat healthy food, the drivers cited many barriers to adopting this behaviour on the road such as limited access, time constraints and the high cost of maintaining a healthy lifestyle whilst travelling [8]. Other factors inhibiting a healthy lifestyle include excessive non-driving work time spent in areas where there are scarce opportunities to purchase healthy food [10], lack of opportunity to seek out food options and engage in physical activity opportunities due to responsibility for cargo in the vehicle [11], lack of suitable parking for larger vehicles in healthy eating zones [7, 8] and availability of low nutritional value food [7].

Lifestyle choice of the worker population has also been identified as a factor influencing the health and wellbeing of truck drivers. Research has found that a current smoking habit was more prevalent in long-haul truck drivers than the general United States (U.S.) working population [12]. Systematic review research found tobacco use among heavy vehicle drivers ranged from 31.5% to 54.9% [13]. Other unhealthy lifestyle factors reported in truck drivers included alcohol misuse [14], drug use [15], obesity [12], and excessive levels of stress [16]. Sleep issues have also been reported for truck drivers due to the health impacts of shift work [17].

These lifestyle and environmental factors have been found to be associated with the development of specific medical conditions such as hypertension [10, 18, 19] and diabetes [20]. Significant cardiovascular risk factors have been reported amongst long-haul truck drivers from analyses of blood samples [21]. Other research has identified truck drivers reporting significant incidences of hypertension, diabetes mellitus, cardiovascular disorders and sleep disorders [22]. A cross-sectional study reported significant incidences of hypertension, diabetes mellitus, and cardiovascular disorders during routine driver fitness examinations of more than 95,000 commercial drivers [22]. Several other studies have found higher rates of hypertension and cardiovascular risk factors in truck drivers compared to the general population [21, 23]. Another sample of long-haul truck drivers, found increased risk of a range of pre-hypertensive conditions, as well as a higher rate of diagnosed diabetes compared to the U.S. adult working age population [18]. In a study of commercial truck drivers, which was controlled for age, it was found that drivers with uncomplicated diabetes not treated with insulin had an increased crash risk compared to other truck drivers [20]. The researchers questioned whether a resulting condition of hypoglycaemia may increase crash risk [20].

These studies suggest that preventative healthcare measures need to be taken to reduce the rate of injury and disease in the industry. However, truck drivers experience challenges to accessing much needed healthcare. To illustrate, the mobile workplace of Australian truck drivers has been identified as a significant barrier to engaging in health interventions [24]. In support, research from the U.S. has found that truck drivers were twice as likely to delay or not utilise necessary health care, compared to the general working population [12]. Another U.S. survey found that almost half of long-haul truck drivers did not have a regular healthcare provider, and almost a third were not able to access needed health services within the previous 12 months [25]. These studies suggest that accessibility to health services may be a factor inhibiting health promotion in some countries.

Attitudinal factors within male-dominated industries may also inhibit access to healthcare for this population of workers. To illustrate, in Australia, despite having a workers’ compensation system, men, overall, access health care less frequently than women, and seek treatment at later stages for a health condition [26]. Men also visit general practitioners less often, have shorter consultations, and raise only one health issue per visit [26]. Past research has found that around 16% of males did not access any government funded healthcare services in an entire year; further to this is that men had less GP encounters than women, yet more emergency department presentations [27]. Attitudinal factors related to accessing health care presents a key issue, considering that the road transportation industry is a large employer of men in Australia, employing 143,710 drivers in 2016, i.e., 2.6 percent of the male workforce [28].

There are multiple factors to consider in facilitating the engagement of health promotion for workers in the transportation industry. To inform the development of feasible and practicable prevention activities, it is firstly important to understand the medical history of truck drivers leading up to an injury, including their pre-injury health and health service use. This information will provide the necessary knowledge to inform secondary and tertiary injury prevention of at-risk truck drivers, including promotional measures such as health screening, monitoring and education.

### Purpose of the study

The aims of this study are to: (i) describe the health and lifestyle behaviour of truck drivers prior to experiencing a workers’ compensation claim for injury, (ii) compare data on injured truck drivers who were admitted to a hospital for a health or lifestyle condition in the five years prior to a workers’ compensation claim in Victoria to other occupational drivers and workers in other industries; and (iii) identify if truck drivers with a workplace injury had an increased likelihood of having previously experienced a health or lifestyle condition which required attention at a hospital five years prior to their injury.

## Method

### Data sources

The Compensation Research Database (CRD) was established by the Institute for Safety Compensation and Recovery Research (ISCRR) at Monash University in 2009 and comprised administrative data from workplace injuries and illnesses that resulted in a compensation claim to WorkSafe Victoria (WSV) since 1985 [29]. WSV acts as the state’s health and safety regulator, and also as the manager of Victoria’s workers (no-fault) compensation scheme; WSV has taken over management of the CRD [30].

The Victorian Admitted Episodes Dataset (VAED) is a compilation of demographic, administrative and clinical data on all admitted patient episodes of care provided by public and private hospitals, rehabilitation centres, extended care facilities and day procedure centres in Victoria [31]. The dataset is maintained by the Victorian Government Department of Health and Human Services (DHHS) Health Data Standards and Systems (HDSS) unit for morbidity monitoring, casemix-based funding and analysis purposes in accordance with several healthcare reporting agreements. Diagnosis data are coded in accordance with the Australian Coding Standards using the ICD-10-AM health classification system (Australian modified version of the current World Health Organisation’s International Classification of Diseases) [32].

### Case selection

Claims data (with injury onset in 2008/09) of truck drivers per ANZSCO classification (7331) and other occupational drivers (the various classifications are listed below) were compared to the compensation claims data of all other (non-driver) Victorian workers. For analysing pre-injury health, cases were only selected if they also had a hospital-recorded admission within five years prior to their injury (based on the *injury onset date* recorded in their workers’ compensation claim). The age of the workers selected for analysis were limited to those over 18 years, due to driving being the focus of the study.

The Australian and New Zealand Standard Classification of Occupations (ANZSCO) code for truck drivers is 733111 [33]. The ‘other occupational driver’ category included the ANZSCO occupational codes of automobile and taxi drivers (731,199, 73,112), bus and coach drivers (731,211, 731,212, 731,213), train drivers (731,311), tram drivers (731,312), and delivery drivers (732,111) [33].

### Data linkage

Research data was sourced via a data linkage method, linking workers’ compensation claims for injury with hospital admissions data. WorkSafe Victoria compensation claims data were sourced from the Institute for Safety Compensation and Recovery Research (ISCRR) Compensation Research Database (CRD) [29]. Data linkage was conducted by the Centre for Victorian Data Linkage (CVDL) located at the Victorian Department of Health and Human Services (currently Department of Health). The CVDL linked the WorkSafe claims data with hospital admissions data, specifically the Victorian Admitted Episodes Dataset (VAED). The data used in the study captured all claims made in 2008/09 (based on affliction year). The hospital admissions data included five years’ pre-injury data relating to these claimants.

### Variables

#### Hospital admissions data

A range of health status variables, lifestyle-related conditions and chronic diseases were selected from the Victorian hospital admissions database if they appeared anywhere in the patient’s record, which can include up to 40 diagnosis-related codes. The group coding for the selected health conditions and chronic diseases was determined from various sources including peer-review publications, government health reports, as well as refinements and inclusions made by the authors [34–38]. Diseases arising from the cardiovascular system have long been implicated as a concern amongst professional drivers [18, 21, 23, 39, 40]. Cardiovascular-related conditions included in this analysis include: atrial fibrillation, chronic pulmonary disease, hypertension, myocardial infarction, peripheral vascular disease, and stroke/transient ischemic attack. The irregular nature of professional driving has also been implicated in contributing to other health factors such as those relating to sleep [21], as well as to diabetes [11]. In addition, several lifestyle concerns have been associated with the occupation of professional driving such as an increased rate of smoking, alcohol and drug use [14, 15, 41, 42], as well as higher incidences of stress and obesity [12, 16, 41]. These health and lifestyle conditions and chronic diseases are captured in the recorded ICD-10-AM diagnosis codes in the Victorian hospital admissions database. Some chronic conditions such as hypertension, diabetes, or depression may not be captured in the hospital admissions records if they were considered not relevant to the admission.

The category codes included in the current study are: atrial fibrillation (ICD-10-AM code I48), chronic pulmonary disease (I27.8, I27.9, J40 – J44, J46 – J47, J60 – J67, J68.4, J70.1, J70.3), diabetes (E10 – E14), hypertension (I10 – I13, I15), myocardial infarction (all types) (I21 – I22, I25.2), peripheral vascular disease (I70 – I71, I73, I77.1, I79.0, I79.2, K55.1, K55.8, K55.9, Z98.8, Z95.9), sleep disorders (G47) and stroke or transient ischemic attack (G45.0 – G45.3, G45.8 – G45.9, H34.1, I60 –I61, I63 – I64). 

The category codes related to lifestyle conditions included in the current study are: alcohol misuse/abuse (F10, E24.4, E51.2, E52, G31.2, G40.5, G62.1, G72.1, I42.6, K29.2, K70, K85.2, K86.0, O35.4, R78.0, T51, X45, X65, Y15, Y90, Y91, Z04.0, Z50.2, Z71.4, Z72.1, Z86.41), drug use/abuse (F11 – F16, F18, F19, X41, X42, X61, X62, Y11, Y12, T40, T42.3, T42.4, T42.6, T42.7, T43.3, T43.5, T43.6, T43.8, T43.9, R78.2 – R78.5, Z50.3, Z71.5, Z72.2, Z86.42), obesity (E66), stress (F43, Z73.3, R45.7), and tobacco use (F17, T65.2, Z50.8, Z58.7, Z72.0, Z71.6, Z81.2,Z86.43).

### Workers compensation claims data

Workers’ details included in the analysis were: gender; age at time of injury; age group at time of injury; Australian and New Zealand Standard Classification of Occupations (ANZSCO) occupation type [33]; Accessibility/Remoteness Index of Australia (ARIA) [43] based on workers’ postcodes and recoded into variables of metropolitan/non metropolitan; and Index of Relative Socio-Economic Advantage and Disadvantage (IRSAD) [44], and coded by state percentile as well as by state decile. Decile 10 represents the most advantaged population-based decile on a scale of 1 to 10 [44].

Employer details featured included the size of the organisation (small, medium, large) or whether it was a government workplace. Employee details were also captured including total weekly earnings pre-injury, and total hours worked per week pre-injury. Details of the workplace injury included in the study were ‘Mechanisms of Injury’ and ‘First Body Location of Injury’.

### Data analysis

Retrospective analysis of information collected in Victoria, Australia, comprised work-related injury data recorded over a one-year period in addition to pre-injury hospital admissions data recorded over a five-year period. Data extraction and preparation was carried out using SAS 9.4 [45] and the descriptive analyses and modelling were carried out using IBM SPSS Statistics 25 [46]. Binary logistic regression was conducted in SPSS to predict outcomes (i.e. disease prevalence, and harmful lifestyle factors) amongst truck drivers versus other occupational drivers, as well as versus all other workers. The model was adjusted for socio-demographic factors such as age, work factors and geographic region. Binary logistic regression was performed on a series of dependent variables including atrial fibrillation, chronic pulmonary disease, diabetes, hypertension, myocardial infarction, peripheral vascular disease, sleep disorders, stroke/transient ischemic attack; in addition to the lifestyle variables of alcohol misuse/abuse, drug misuse/abuse, obesity, stress and tobacco use. The independent variables were occupation (truck driver/other occupational driver/non-driver), injury age, weekly earnings, weekly hours worked, ARIA (metropolitan/non-metropolitan) and IRSAD.

## Results

### Descriptive data

In total, 45,646 claims for compensation by Victorian workers aged over 18 years were included in the initial analysis. These were claims in which a worker experienced a workplace injury or disease in the year of 2008/09 and subsequently claimed compensation through WorkSafe Victoria. Table 1 displays the data summary of age, gender, IRSAD, ARIA and employment characteristics for the 45,646 workplace claims. The most common age group for truck drivers with a workers’ compensation claim was the 45 to 54-year old age group (30% of all truck drivers). In regards to gender, females were of the minority of cases in all categories: truck drivers (2.0%), other occupational drivers (10.9%), and all other workers (36.1%). For the Index of Relative Socio-economic Advantage and Disadvantage, truck drivers constituted only 9.8% of Decile 9 and 10 (which are the most advantaged groups) compared to all other workers at 20.0%. Truck drivers with workplace injuries are also more likely to live in a regional or remote area (37.8%) compared to other claimants (28.5%).

**Table 1:** Descriptive Statistics of the Dataset

	**Truck Drivers**	**Other Occupational Drivers**	**All Other Claimants**	**Total**
Count (Total of All Claims)	1712	1115	42,819	45,646
Average Injury Age (years), [min, max]	45.7 [18, 78]	46.4 [18, 76]	41.5 [18, 99]	44.5 [18, 99]
18 to 24 Years	45 (2.6%)	47 (4.2%)	5217 (12.2%)	5309 (11.6%)
25 to 34 Years	242 (14.1%)	141 (12.6%)	8389 (19.6%)	8772 (19.2%)
35 to 44 Years	492 (28.7%)	264 (23.7%)	10,228 (23.9%)	10,984 (24.1%)
45 to 54 Years	512 (29.9%)	366 (32.8%)	11,484 (26.8%)	12,362 (27.1%)
55 to 64 Years	368 (21.5%)	264 (23.7%)	6772 (15.8%)	7404 (16.2%)
65 Plus Years	53 (3.1%)	33 (3.0%)	729 (1.7%)	815 (1.8%)
Males	1678 (98.0%)	994 (89.1%)	27,349 (63.9%)	30,021 (65.8%)
Females	34 (2.0%)	121 (10.9%)	15,470 (36.1%)	15,625 (34.2%)
IRSAD State Decile 1 and 2	435 (25.5%) *	218 (19.6%) *	8558 (20.0%) *	9211 (20.2%) *
IRSAD State Decile 3 and 4	434 (25.4%) *	214 (19.2%) *	7903 (18.5%) *	8551 (18.8%) *
IRSAD State Decile 5 and 6	366 (21.4%) *	259 (23.3%) *	9218 (21.6%) *	9843 (21.6%) *
IRSAD State Decile 7 and 8	304 (17.8%) *	234 (21.0%) *	8518 (19.9%) *	9056 (19.9%) *
IRSAD State Decile 9 and 10	168 (9.8%) *	188 (16.9%) *	8562 (20.0%) *	8918 (19.6%) *
*% of valid data	[= 1707*]	[= 1113*]	[= 42,759*]	[= 45,579*]
ARIA Major Cities	1063 (62.2%) *	865 (77.9%) *	30,564 (71.5%) *	32,492 (71.4%) *
ARIA Inner/Outer Regional/Remote	646 (37.8%) *	246 (22.1%) *	12,153 (28.5%) *	13,045 (28.6%) *
*% of available and valid data	[= 1709*]	[= 1111*]	[= 42,717*]	[= 45,537*)

Of the initial 45,646 claims, there were 22,528 Victorian workers who additionally had at least one recorded Victorian hospital admission within five years prior to their injury claim date; these claims constitute the main sample for analysis in the study. The sample was divided into injured worker groups of: 1) truck drivers, 2) other occupational drivers, and 3) workers in other occupations (i.e., non-drivers). Analysis focused on a comparison between these groups. Table 2 illustrates the breakdown of claims data for each occupational group. Please refer to Table 2 for further clarification regarding details of Tables 5, 6, 7 and 8.

**Table 2:** Summary of Claims Data Incorporated in the Analyses

	Truck Drivers	Other Occupational Drivers (excluding Truck Drivers)	All Other Occupational Claimants
Total Claims (Initial Analysis)	1712	1115	42,819
Claims with corresponding Hospital Admission (≥ 1) with 5 Years of Injury (Main Analysis)	822	489	21,217

### Employer data

Almost one third of employee claims by truck drivers (30.0%) were from a small-sized employer (i.e., less than $1 million remuneration 2010/11) compared to 19.4% for the non-driver claimant group (Table 3). Conversely, large and government-based employer claims were less common among the truck driver workplace claims (19.8%) compared to 39.1% for non-driver claimants and 45.2% of other occupational drivers. Among the claimants, truck drivers had a higher number of pre-injury hours worked per week (mean: 35.6 h) compared to other occupational drivers (mean: 34.1 h) and non-driver claimants (mean: 32.9 h).

**Table 3:** Pre-Injury Employment Characteristics

Pre-Injury Descriptive Information	Truck Drivers *n* = 1712	Other Occupational Drivers *n* = 1116	Non-Drivers *n* = 43,266
	Hours	Hours	Hours
Ordinary Hours Worked (Mean)	35.6	34.1	32.9
Size of Employer	n (%)	n (%)	n (%)
Small	513 (30.0%)	299 (26.8%)	8458 (19.5%)
Medium	859 (50.2%)	313 (28.0%)	17,879 (41.3%)
Large/Government	340 (19.8%)	504 (45.2%)	16,929 (39.1%)

A (low) default value is entered routinely for minor claims where earnings of the worker have not been verified. Therefore, only pre-injury earnings for standard claims are calculated. These were (mean) AU$697 for truck drivers, AU$599 for other occupational drivers, and $628 for other workers.

### Workplace injury data

In regards to the type of workplace injury (Table 4), in order of prevalence, the top five mechanisms of injury reported by truck drivers were: body stressing; falls, trips, slips; being hit by moving objects; vehicle incidents; and hitting objects with a part of the body. The top five injury mechanisms for other occupational drivers were: body stressing; vehicle incidents; falls, trips, slips; being hit by moving objects; and mental stress. For non-driver claimants, the top five mechanisms of injury were: body stressing; vehicle incidents; falls, trips, slips; being hit by moving objects; and mental stress.

**Table 4:** Mechanisms of Workplace Injury

	Highest prevalence				
	Rank 1	Rank 2	Rank 3	Rank 4	Rank 5
Truck drivers	Body stressing	Falls, trips, slips	Being hit by moving object	Vehicle incidents	Hitting incidents with a part of the body
Other occupational drivers	Body stressing	Vehicle incidents	Falls, trips, slips	Being hit by moving object	Mental stress
All other claimants	Body stressing	Falls, trips, slips	Being hit by moving object	Hitting incidents with part of body	Mental stress

### Comparison of truck drivers to other occupational drivers

Logistic Regression modelling was applied to investigate pre-injury health and lifestyle factors in truck drivers who subsequently made a claim for compensation when compared to (i) all other occupational drivers who made a claim for workers compensation and (ii) all injured workers. The results of the driver group subset of 1,311 cases (derived from the main analysis) are displayed in Table 5 and Table 6. This analysis only included those that had at least one hospital admission in the five years prior to their workplace injury. The models were adjusted for injury age, total weekly earnings, hours worked per week, ARIA, and IRSAD state percentile. After adjustment of these factors, truck drivers were found to have greater likelihood of having a hospital-recorded health condition of diabetes, and hypertension prior to a workplace injury when compared to other occupational drivers (Table 5). Compared to other occupational drivers, truck drivers were less likely to have a pre-affliction hospital-recorded sleep disorder. In addition, truck drivers had a greater likelihood of having a hospital-recorded lifestyle factor of alcohol misuse/abuse and tobacco use prior to a workplace accident when compared to other occupational drivers (Table 6).

**Table 5:** Logistic Regression of Truck Drivers (*n* = 822) Compared to Other Occupational Drivers (*n* = 489) (Health Conditions)

**Dependent Variable**	**Atrial Fibrillation**		**Chronic Pulmonary Disease**		**Diabetes**		**Hypertension**	
**Subset of Cases (Only Truck Drivers & Other Occupational Drivers) = 1311**								
**Independent Variable**	**p**	**Odds Ratio**	**p**	**Odds Ratio**	**p**	**Odds Ratio**	**p**	**Odds Ratio**
Truck Driver	0.291	1.583	0.977	0.980	**0.011**	1.872	**0.022**	1.715
Injury Age	**0.000**	1.088	0.117	1.055	**0.000**	1.074	**0.000**	1.092
Weekly Earnings	0.814	1.000	0.258	0.999	0.494	1.000	0.707	1.000
Work Hours / Week	0.284	0.977	0.063	0.999	0.244	0.985	0.504	0.991
Accessibility Remoteness	0.754	0.878	0.244	2.716	0.249	1.317	**0.025**	1.742
IRSAD State Percentile	0.183	0.990	0.272	0.986	**0.000**	0.985	**0.013**	0.990
	**Myocardial Infarction**		**Peripheral Vascular Disease**		**Sleep Disorder **		**Stroke or Transient Ischemic Attack**	
	**p**	**Odds Ratio**	**p**	**Odds Ratio**	**p**	**Odds Ratio**	**p**	**Odds Ratio**
Truck Driver	0.504	1.262	0.520	0.691	**0.014**	0.511	0.075	3.961
Injury Age	**0.001**	1.059	**0.005**	1.097	0.481	1.009	0.111	1.044
Weekly Earnings	0.128	1.001	0.460	1.000	0.700	1.000	0.463	1.000
Work Hours / Week	0.687	0.991	**0.038**	1.089	0.454	1.014	0.253	0.966
Accessibility Remoteness	**0.018**	3.028	0.255	2.508	0.814	1.076	0.714	1.242
IRSAD State Percentile	0.842	0.999	0.770	0.997	0.819	0.999	0.453	0.992

**Table 6:** Logistic Regression of Truck Drivers (*n *= 822) Compared to Other Occupational Drivers (*n* = 489) (Lifestyle Conditions)

Dependent Variable Subset of Cases	Alcohol Misuse/Abuse		Drug Misuse/Abuse		Obesity		Stress		Tobacco Use	
Subset of Cases (Only Truck Drivers & Other Occupational Drivers) = 1311										
Independent Variable	p	Odds Ratio	p	Odds Ratio	p	Odds Ratio	p	Odds Ratio	p	Odds Ratio
Truck Driver	**0.046**	1.989	0.835	1.085	0.383	0.724	0.296	2.000	**0.000**	1.592
Injury Age	0.054	0.974	**0.000**	0.929	0.574	1.010	**0.025**	0.948	**0.002**	1.017
Weekly Earnings	0.114	1.001	0.190	1.001	0.511	1.000	0.169	1.001	0.380	1.000
Work Hours / Week	0.189	0.976	0.101	0.964	0.785	0.994	0.952	1.002	0.460	1.005
Accessibility Remoteness	0.146	1.696	0.383	1.460	0.822	1.092	0.305	0.567	**0.019**	0.744
IRSAD State Percentile	0.459	1.004	0.664	0.997	**0.022**	0.983	0.712	1.004	0.654	0.999

### Comparison of truck drivers to all injured workers

Logistic Regression modelling was applied to investigate the incidence of health and lifestyle factors in truck drivers who subsequently made a claim for compensation when compared to all other workers who made a compensation claim. This analysis utilised a set of 22,528 claims in the main analysis. The analysis only included those that had at least one hospital admission five years prior to their workplace injury. After adjustment of socio-demographic factors such as age, work-related factors and geographic region, truck drivers had a greater likelihood of having a hospital-recorded health condition of atrial fibrillation, diabetes, hypertension, myocardial infarction, and stroke/transient ischemic attack prior to a workplace accident when compared to all other workers with compensation claims (Table 7). In addition, truck drivers had a greater likelihood of having a hospital-recorded lifestyle factor of alcohol misuse/abuse and tobacco use prior to the affliction, when compared to all other workers with compensation claims (Table 8).

**Table 7:** Logistic Regression of Truck Drivers (*n* = 822) Compared to All Other Claimants (*n *= 21,217) (Health Conditions)

**Dependent Variable**	**Atrial Fibrillation**		**Chronic Pulmonary Disease**		**Diabetes**		**Hypertension**	
**All Cases**								
**Independent Variable**	**p**	**Odds Ratio**	**p**	**Odds Ratio**	**p**	**Odds Ratio**	**p**	**Odds Ratio**
Truck Driver	**0.006**	1.948	0.921	0.955	**0.000**	2.064	**0.000**	1.870
Injury Age	**0.000**	1.083	**0.000**	1.043	**0.000**	1.063	**0.000**	1.091
Weekly Earnings	0.281	1.000	0.098	1.000	0.249	1.000	0.300	1.000
Work Hours / Week	0.137	1.012	0.920	1.001	0.866	1.001	0.968	1.000
Accessibility Remoteness	0.623	0.924	0.545	0.881	**0.030**	1.199	**0.028**	1.199
IRSAD State Percentile	0.695	1.001	0.055	0.993	**0.000**	0.992	**0.000**	0.993
	**Myocardial Infarction**		**Peripheral Vascular Disease**		**Sleep Disorder**		**Stroke or Transient Ischemic Attack**	
	**p**	**Odds Ratio**	**p**	**Odds Ratio**	**p**	**Odds Ratio**	**p**	**Odds Ratio**
Truck Driver	**0.001**	2.007	0.273	1.553	0.194	1.295	**0.009**	2.239
Injury Age	**0.000**	1.084	0.000	1.108	**0.000**	1.038	**0.000**	1.072
Weekly Earnings	0.177	1.000	0.009	0.999	**0.004**	1.000	0.072	1.000
Work Hours / Week	**0.005**	1.021	0.039	1.026	0.249	0.994	0.282	0.990
Accessibility Remoteness	0.076	1.305	0.484	0.842	**0.000**	1.563	0.686	0.919
IRSAD State Percentile	**0.011**	0.994	0.287	0.996	0.504	1.001	0.500	0.998

**Table 8:** Logistic Regression of Truck Drivers (*n* = 822) Compared to All Other Claimants (*n *= 21,217) (Lifestyle Conditions)

Dependent Variable	Alcohol Misuse/Abuse		Drug Misuse/Abuse		Obesity		Stress		Tobacco Use	
All Cases										
Independent Variable	p	Odds Ratio	p	Odds Ratio	p	Odds Ratio	p	Odds Ratio	p	Odds Ratio
Truck Driver	**0.000**	1.885	0.087	1.494	0.282	1.303	0.203	1.466	**0.000**	1.808
Injury Age	**0.000**	0.970	**0.000**	0.954	**0.001**	1.015	**0.000**	0.969	**0.000**	1.009
Weekly Earnings	0.113	1.000	0.278	1.000	0.048	1.000	0.540	1.000	0.437	1.000
Work Hours / Week	0.369	0.996	**0.023**	0.988	**0.000**	0.980	**0.020**	0.985	0.471	0.999
Accessibility Remoteness	0.099	1.177	**0.013**	1.331	0.204	1.173	0.347	0.873	**0.000**	0.786
IRSAD State Percentile	**0.000**	0.993	**0.000**	0.993	**0.017**	0.995	0.447	0.998	**0.000**	0.994

## Discussion

The aim of this study was to describe the health and lifestyle behaviour of truck drivers prior to experiencing a workers’ compensation claim for injury, and then compare the data to other occupational drivers, and to other workers across other industries. The study then sought to uncover any health and lifestyle factors that may pre-dispose a truck driver to workplace injury. This study extends the findings of previous research in Australia showing that truck drivers are at significant risk of work injury claims [2, 47]. This study found that when compared to all other workers’ compensation claimants, truck drivers had a greater likelihood of having a hospital record of alcohol misuse/abuse, tobacco use, atrial fibrillation, diabetes, hypertension, myocardial infarction, and/or stroke/transient ischemic attack prior to their workplace incident. This study also found that when compared with other occupational drivers, truck drivers had a greater likelihood of having a hospital-recorded health condition of diabetes and/or hypertension, and lesser likelihood of a hospital-recorded sleep disorder in the five-year period leading up to a workplace accident. Truck drivers also had a greater likelihood of having a hospital-recorded diagnosis code for alcohol misuse/abuse and tobacco use prior to a workplace incident when compared to other occupational drivers. These results have implications for the review and revision of control measures, as well the development of new controls, to promote the health and wellbeing of truck drivers.

### Health factors

Compared to all other workers with an occupational injury, the current study found that truck drivers with an occupational injury had an increased risk of having atrial fibrillation, diabetes, hypertension, myocardial infarction, and/or stroke/transient ischemic attack prior to their workplace incident. In addition, the current study found that compared to other occupational drivers, truck drivers had an increased risk of having diabetes and hypertension prior to their workplace incident.

The current study findings are in agreement with other published studies that have reported significant incidences of hypertension, diabetes mellitus, and cardiovascular disorders amongst professional drivers [18, 21–23]. Longer working hours are associated with an increased likelihood of having hypertension [48] which may help to explain the increased risk in truck drivers compared to other professional drivers. A recommendation of the current study is that workplaces integrate health and wellbeing programs as part of their occupational health and safety risk management strategies to not only screen for these conditions but to monitor employees over time. This approach being particularly important for ageing drivers [3, 49, 50]. In support, it has been found that the workplace is an effective medium for screening diabetes, and for improving health outcomes for diabetics one year later [51]. The workplace is also an effective setting to detect pre-diabetic cases [52]. As truck drivers are not regular consumers of health care services [25], the workplace would be the ideal place to locate drivers at risk of diabetic complications that may impact their driving ability. A diabetes prevention screening program has been shown to reduce absenteeism in the workplace after two years [53]. Workplace screening programs are also effective for identifying employees with undiagnosed and/or untreated hypertension [54]. A workplace screening program in Germany resulted in around 75% of workers with detected hypertension following up with medical management of their condition [55]. Additionally, a hypertension screening program aimed at commercial drivers found cost savings in terms of worker productivity after two years [56].

In regards to truck drivers being less likely to have a sleep disorder than other occupational drivers, this finding is not consistent with other studies that report a high prevalence of sleep disorders amongst truck drivers [21, 57, 58], although many of these studies are conducted in North America. In Australia, truck drivers are given a ‘Fitness to Drive’ medical examination prior to obtaining a commercial driver’s licence and/or in some states on licence renewal if the driver is an older driver; part of the examination is a sleep apnoea assessment featuring a measurement tool of sleepiness [59]. The nature of the driving task is taken into consideration when granting a commercial licence, with a more stringent assessment given to full-time drivers of heavy vehicles on interstate highways with multiple combination trailers [59].

### Lifestyle factors

The current study found that, compared to all other workers with occupational injuries, truck drivers had a greater likelihood of having an association with tobacco use. Furthermore, when compared to other occupational drivers only, truck drivers were still more likely to have a hospital-recorded history of tobacco use, after adjusting for factors such as age, sex and socio-economic status (SEIFA). Other studies have also identified a higher incidence of tobacco use amongst truck drivers compared to the general population [12, 21, 23, 41]. In another study of smoking amongst various occupational professions, long-haul truck drivers had the highest rate of smoking, with 18% being current smokers and 49% being former smokers [60].

The current study found that truck drivers with a compensation claim had a higher likelihood of having a hospital-recorded concern of alcohol use/misuse prior to their workplace injury when compared to other professional drivers with a compensation claim, as well as compared to all other workers with a compensation claim. A meta-analyses of alcohol consumption worldwide amongst truck drivers found that 19% engaged in regular patterns of binge drinking, whilst there was an ‘everyday drinking’ pattern of 9.4% [14]. These findings suggest that the driving context, especially long-haul, may predispose drivers to engage in these unhealthy behaviours. Understanding the reasons for this use is important in informing prevention activities. A review of workplace prevention and intervention alcohol programs found some success can be obtained by using a combination of education, counselling, web-based and brief intervention strategies [61]. Workplace programs directed at quitting smoking have also been demonstrated to be successful, with counselling and pharmacological treatment being most effective [62].

In summary, in support of previous research [4, 5], prevention activities could focus at multiple levels of the transportation system including: (i) the workplace, using health promotion strategies, as well as health education programs; (ii) via regulation, through supporting employers in developing guidance material and the tools to help employers to target risk; and (iii) with government, e.g. through initiatives to upskill medical practitioners in identifying health issues based on occupational grouping. Even with the occupational resources of a large employer, many truck drivers face barriers to health promotion participation from their limited access to employer services due to being out on the road. Mobile health services, including screening clinics, and financial support from governments could help alleviate these barriers to health promotion participation in the workplace. Additionally, preventative health campaigns and encouraging healthy lifestyle discussions with health professionals could also be beneficial to truck drivers [27].

### Strengths & limitations

The strength of this study is that the data collected is generalisable as it was collected on adults with a workers’ compensation claim across all industry occupations and featured a wide range of afflictions. This study also overcame the bias inherent in previous research using self-report measures or self-selection into the sample. Despite these strengths, bias towards a less healthy population may exist. That is, past research has compared truck drivers to the general population, as opposed to comparing them to other injured workers with compensation claims, as was undertaken in this study. This study also analysed data for those with a hospital admission prior to their workplace injury and thus may represent a less healthy population. It is also likely that chronic health conditions and lifestyle problems were not fully, systematically recorded in the hospital admissions database, as they were only included if they were relevant to that particular episode of care. Thus, the data may underestimate the prevalence of some conditions. A further limitation of the study is that it included data from the financial year, 2008 to 2009. The current study is a small component of a broader research program conducted on linkage data that originally analysed pre-injury conditions (5 years) and post injury outcomes (7 years after) for a cohort of Victorian WorkSafe claimants with associated afflictions occurring in the 2008/09 financial year [63].

## Conclusion

This study found that truck drivers were more likely to experience specific health and lifestyle conditions prior to a workplace injury than other occupational drivers. Five years preceding a workplace incident, truck drivers admitted to a Victorian hospital (for any reason) were more likely to have a hospital-recorded medical condition of diabetes or hypertension when compared to other occupational drivers. Additionally, this study found that prior to a workplace injury claim, truck drivers with at least one hospital admission record were more likely to have hospital-recorded tobacco use and/or alcohol misuse/abuse when compared to other occupational drivers. These results have implications for the review and revision of existing health strategies, as well as the development of new interventions, to promote the health and wellbeing of truck drivers. The results support the need for engagement of the employers, regulators and government bodies in implementing and promoting health screening which targets the identified lifestyle behaviours. Additionally, it would be useful to raise awareness among medical practitioners with regard to the importance of identifying health issues based on occupational grouping, as well as ensuring any medical conditions are well managed.

## Data Availability

Fully de-identified workers' compensation claims data may be available upon request, either by contacting WorkSafe directly or by contacting the Institute for Safety, Compensation and Recovery Research (ISCRR). However, this will be dependent on ethical clearance as well as data custodian approval.
